# Supplementary material for: Lithium ions solvated in helium
Source: Phys Chem Chem Phys. 2018 Aug 8;20(40):25569–76. doi: 10.1039/c8cp04522d (PMC6194493; doi:10.1039/c8cp04522d)
Supplement: Supplementary file 1 [file CP-020-C8CP04522D-s001.pdf]

Electronic Supplementary Material (ESI) for Physical Chemistry Chemical Physics.
This journal is © the Owner Societies 2018

Supporting Information: Lithium ions solvated in helium

M. Rastogi[a], C. Leidlmair[a], L. An der Lan[a], J. Ortiz de Zárate[b], R. Pérez de Tudela[c], M. Bartolomei[b], M. I. Hernández[b], J. Campos-Martínez[b], T. González-Lezana[b]*, J. Hernández-Rojas[d], J. Bretón[d], P. Scheier[a], and M. Gatchell[a,e]*

[a]Institut für Ionenphysik und Angewandte Physik, Universität Innsbruck, Technikerstr. 25, A-6020 Innsbruck, Austria
[b]Instituto de Física Fundamental, IFF-CSIC, Serrano 123, 28006 Madrid, Spain
[c]Lehrstuhl für Theoretische Chemie, Ruhr-Universität Bochum, 44780 Bochum, Germany
[d]Departamento de Física and IUdEA, Universidad de La Laguna, 38205 Tenerife, Spain
[e]Department of Physics, Stockholm University, 106 91 Stockholm, Sweden

July 17, 2018

*t.gonzalez.lezana@csic.es
*michael.gatchell@uibk.ac.at

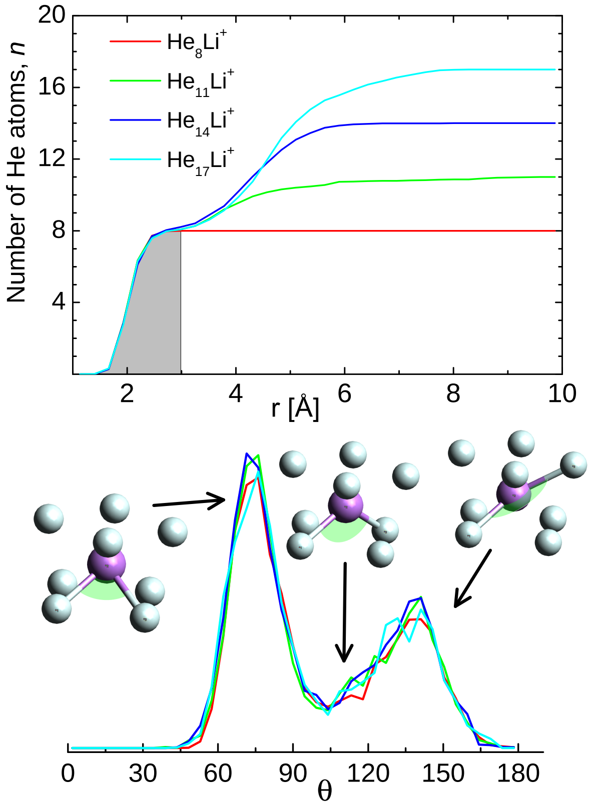

Figure 1: (top) Number of He atoms inside a sphere of radius r around the Li^+ ion in He_nLi^+ droplets with $n = 8$, 11, 14 and 17. The first solvation layer around the ionic impurity is formed by 8 He atoms as shown in the above panel with all distributions sharing the area in shadow covering up to $r \sim 3$ Å. Further confirmation is observed in the angular distribution in the bottom panel where the same pattern with the three features for the corresponding He–Li^+–He angles in the He_2–Li^+ clusters is found for the $n = 11$, 14 and 17 cases.
